# Impact of an on-line educational program on life support treatment limitation (lstl) and potentiality for donation after controlled cardiac death (cdcd) of critically ill patients in 11 hospitals of catalonia

**DOI:** 10.1186/2197-425X-3-S1-A652

**Published:** 2015-10-01

**Authors:** A Sandiumenge, G Moreno, M Llauradó, N Masnou, E Oliver, M Ibañez, M Lopez, B Cancio, E Navas, G Miró, M Jurado, M Badia, MD Bosque Cebolla, J Twose, T Pont, M Bodi

**Affiliations:** University Hospital Vall d´Hebron, Barcelona, Spain; University Hospital Joan XXIII, Tarragona, Spain; University Rovira i Virgili, Tarragona, Spain; University Hospital Josep Trueta, Girona, Spain; University Hospital Bellvitge, Barcelona, Spain; University Hospital Virgen de la Cinta, Tortosa, Spain; Hospital de Vic, Vic, Spain; University Hospital Moises Broggi, Barcelona, Spain; University Hospital Mutua de Tarrasa, Tarrasa, Spain; Hospital de Mataró, Mataró, Spain; Hospital de Tarrasa, Tarrasa, Spain; University Hospital Arnau de Vilanova, Lleida, Spain; Hospital General de Cataluña, Barcelona, Spain; OCATT, Barcelona, Spain; University Hospital Joan XIII, Tarragona, Spain

## Introduction

The lack of education on LSTL and cDCD of intensive care unit (ICU) health-care professionals may lead to misperceptions and contributes to negative attitudes hampering the development of such programs(1).

## Objectives

We aimed to assess the impact of LSTL and cDCD training on the End-of-life care practices and potentiality for cDCD of 11 catalonian ICUs.

## Methods

Data on End-of-Life Care of critically ill patients admitted to 11 catalonian ICUs was prospectively collected before (P1: 01/3-31/06 2013) and after (P2: 01/2-30/05) an on-line training educational program on LSTL and cDCD delivered to 58 nurses and 62 doctors of the participating centres. Potential for cDCD was assessed through the analysis of clinical, analytical and agonal times (time from LSTL initiation to asystole) of patients in whom withdrawal of mechanical ventilation (MV) and/or vasoactive support (VAS) was performed as a form of LTSL.

## Results

A total of 6616 patients (P1:3315; P2:3301) were admitted with similar rates (P1:9.8%; P2:9.6%) and characteristics of patients undergoing LSTL in both periods. No differences were observed on the time from admission to First (5,19 ± 9.0 vs 4.33 ± 8.94 days) and Definitive-LSTL (D-LSTL-the one preceding patient´s death) (P1:n=215; 6.83 ± 11.6 vs P2:n=205; 6.97 ± 11.0 days) actions between periods. Futility (P1:74%; P2:73%), admission diagnosis (P1:62%; P2:50%) and co-morbidity (P1:40%; P2 45.9%) were the main causes for D-LSTL in both periods. Treatment withdrawal was the most common form of D-LSTL (P1:57.7%; P2:51.2%) with higher rates of ventilator support withdrawal during P2 (80% vs 67%)p < 0.05. Sedation and/or analgesia was provided in 81%(P1) and 82.6%(P2) of patients in whom treatment was withdrawn. Agonal times after treatment withdrawal were shorter in P2(n = 105)(115.0 min (25-75ICR 37.0-405.0) compared to P1(n=124)(197.5 (25-75ICR 55.0-675.0)(p < 0.05). Six (7.7%) and 4(5.5%) patients in whom VAS and MV was withdrawn during P1 and P2 could have been cDCD donors representing a 24% and 25% increase over the DBD donor pool respectively.

## Conclusions

Although not influencing the potentiality of cDCD, training on LSTL improved end-of-life practices on critically ill patients.
